# Delineation of the Prostate Bed: The “Invisible Target” Is Still an Issue?

**DOI:** 10.3389/fonc.2017.00108

**Published:** 2017-05-31

**Authors:** Igor Latorzeff, Paul Sargos, Geneviève Loos, Stéphane Supiot, Stéphane Guerif, Christian Carrie

**Affiliations:** ^1^Department of Oncology Radiotherapy, Bât Atrium, Clinique Pasteur, Toulouse, France; ^2^Department of Radiotherapy, Institut Bergonié, Bordeaux, France; ^3^Department of Radiotherapy, Centre Jean-Perrin, Clermont-Ferrand, France; ^4^Department of Radiotherapy, Institut de Cancérologie de L’Ouest René Gauducheau, Nantes, France; ^5^Department of Radiotherapy, CHU de Poitiers, Poitiers, France; ^6^Department of Radiotherapy, Centre Léon Bérard, Lyon, France

**Keywords:** prostate cancer, postoperative, radiotherapy, volume delineation, clinical target volume

## Abstract

For pathological high-risk prostate cancer, adjuvant irradiation has shown a survival benefit. Phase III studies have highlighted that half men would face biochemical relapse and would be candidate for radiotherapy at adjuvant or salvage times. Despite at least four published international contouring guidelines from different collaborative groups, discrepancies remain for volumes, delineation, and margins to be considered in order to optimize radiotherapy planning. This article from “Groupe d’Etude des Tumeurs UroGénitales (GETUG)” members will focus on controversies to help clinicians to create best volume delineation for adjuvant or salvage post prostatectomy radiotherapy.

## Introduction

Radiotherapy (RT) after radical prostatectomy (RP) is indicated in the adjuvant setting for patients with high-risk pathological features (1) in the salvage setting at prostate-specific antigen (PSA) relapse or when the PSA remains elevated after RP (2). With long-term follow-up, it has been demonstrated that in 40% of patients treated with adjuvant RT who develop a recurrence, the predominant site remains local (3). The potential reasons for local failure include an inadequate radiation dose and inadequate definition of the clinical target volume (CTV). Successful RT in the era of three-dimensional conformal RT (3D-CRT) and intensity-modulated RT (IMRT) requires physicians to accurately delineate treatment targets while simultaneously avoiding normal tissue to limit organ at risk (OAR) toxicity. Four consensus guidelines have been published for CTV delineation in postoperative RT. Significant differences exist between these guidelines with respect to CTV delineation. In the postoperative setting where the macroscopic target volume has been removed completely both the delineation of the CTV and the precision of dose delivery become crucial especially when attempting increasing dose, as IMRT allows it. The use of image-guided radiotherapy (IGRT), to optimize patient positioning for postoperative RT, is increasing and is directly derived from accurate target volume definition and appropriate margins (4). This article will focus on CTV delineation discrepancies with modern 3D conformational radiotherapy based on computed tomography (CT) scan or magnetic resonance imaging (MRI) used for planning.

## Definition of CTV for Prostate Bed

For postoperative RT, gross tumor volume (GTV) does not exist clearly in adjuvant setting and it can be hardly estimated, clinically or radiologically, for salvage purpose in condition of a rising PSA because it remains microscopic most of the time. CTV definition is based from pathological study of the prostate: size of the gland, seminal vesicle (SV) invasion, and location of positive margins (5). This volume corresponds to the prostate bed, and we are going to highlight how challenging is the definition of CTV for the prostate fossa.

### CTV Delineation following Locations of Recurrence after RP

Following RP, the rate of biochemical failure is relatively high, >50%, within the first 5 years among patients with pathological high-risk features (positive surgical margins, extracapsular extension, and SV involvement) (6). To determine the optimal CTV for planning, it is necessary to appreciate the most common sites of local relapse after surgery. In few cases, a local relapse can be confirmed by physical examination, TRUS-guided biopsy, MRI, and sometimes choline positron emission tomography (PET). Some studies describe the site of a biopsy proven relapse in the prostatic bed after prostatectomy. For Silverman and Krebs, all the 31 local clinically detected relapses were located at the vesicourethral anastomosis (VUA) (7) and that was the case for 2/3 of the patients in Connolly study (location anterior, posterior or both) (8). Leventis et al. reported 17/31 positive biopsies at the VUA in a TRUS series of 41 biochemically relapsing patients after RP. Other sites of interest were bladder neck and retrovesical space and residual SV (9). Contrast-enhanced, endorectal coil MRI has a high sensitivity and specificity for detecting local recurrence after RP (10, 11). In a study on 48 patients, Sella et al. showed that local recurrences were perianastomotic in 29% of patients, retrovesical in 40%, in residual SV in 22%, and at surgical margins (anterior or lateral) in 9% (10). In a series by Miralbell et al., MRI was capable of documenting a recurrent or residual disease in the setting of PSA levels ranging from 0.05 to 13.3 ng/mL (median: 0.87), typically in the inferior and posterior region of the vesicourethral anastomosis (11). These results from an MRI series of 60 men are consistent with another MRI study showing recurrences largely around the VUA (12). At last, ^18^F-fluorodeoxyglucose or ^11^C-acetate PET were tested in 20 consecutive patients with suspected residual or recurrent prostate cancer after RP and with PSA levels of <1 ng/mL with PET/CT co-registration, and 5 and 6 local recurrences were identified, respectively, following techniques used (13). On 33 patients with biological and histopathological evidence of recurrence, focally increased [(11)C]choline uptake in the prostatic bed reliably predicted local low volume occult relapsing prostate adenocarcinoma after RP and identified 71% of patients with a favorable biochemical response to local radiotherapy in a study by Reske et al. (14). These results emphasized similarity, whatever diagnosis methods used, in recurrence location that helps to create a CTV on a planning CT scan, even if correlation between anatomically described location and radiographic positioning remain difficult in a postoperative setting. As described in a comparison between pre- and postsurgery planning CT scan by Sanguinetti et al., the positions of bladder and rectum are shifted in the prostate fossa and the volume of CTV is reduced by 30% after surgery following variations of these anatomical strictures (15). To help clinicians to detect regions of interest (ROI), clips placement by the surgeon during prostatectomy could locate anastomosis between bladder neck and urethra and could be used as fiducial markers for IGRT (4, 16). On the other hand, sometimes the risk of compromising ROI delineation could exist with the great numbers of hemostatic surgical clips placed in the prostate fossa as they can also hinder this identification due to the image artifacts that they can cause. Hence, due to the complexity of CTV definition after surgery (due to changes in anatomy caused by the surgery itself and the limited information on the preoperative location of the prostate), consensus and guidelines for prostate bed delineation became crucial.

### Guidelines to Delineate the Prostate Bed

Nowadays, in the literature exists at least four consensus guidelines for postoperative external beam radiotherapy for prostate cancer focusing on CTV consensus guidelines using CT, in the era of 3D-CRT: the European Organization for Research and Treatment of Cancer (EORTC) (17), the Australian and New Zealand Radiation Oncology Genito-Urinary Group [the Faculty of Radiation Oncology Genito-Urinary Group (FROGG-RANZCR)] (18), the Princess Margaret Hospital (PMH) (19), and the Radiation Therapy Oncology Group (RTOG) (20). The CTV definitions based on each consensus are listed and summarized in Table 1. Most of them explore two patients’ cases scenarios (pT2R1 or pT3a as case 1 and pT3b as case 2) to describe best guidelines recommendations. Of note, each working group developed its CTV definition following a limited number of experts gathered in a delineating task group panels. The FROGG consensus was refined during a consensus conference in June 2006 attended by 63 specialists (radiation oncologists, urologists, diagnosis imaging experts) and issues were developed subsequently in working groups to generate the published guidelines (18). For PMH consensus, 3 experienced urologists then 2 radiation oncologists delineated first boundaries contours for CTV (on CT or MRI), and this result was revised and approved during a GU tumor board meeting gathering 15 medical experts, and second, this proposal was validated by 2 radiation oncologists (19). The EORTC panelist board conducted a review of the likelihood of cancer recurrence from literature to publish its final consensus revised by EORTC members (17). Finally, during an RTOG-sponsored meeting, 11 radiation oncologists delineated prostate fossa CTV (pfCTV) on 2 cases (pT2c R1 with rising PSA and pT3bR0 with undetectable PSA) and their results were matched and statistically compared to finally accept a general agreement concordance (20). It is accepted that CTV should encompass the prostate and the SV surgical bed at risk of harboring microscopic disease or involved following pathological features. The planning process should include then preoperative imaging (CT and/or MRI), intraoperative reports and histopathological findings. The CTV delineation was reported on non-contrast CT (except for FROGG guideline) or CT/MRI images for simulation. The four consensus groups also agree that the vesicourethral anastomosis and periurethral tissue should be treated but they highlight discrepancies in including differently surrounding tissues like bladder or SV bed resulting in a great difference of CTV volume as shown in Table 1. The CTV volume from EORTC consensus has been showed to be significantly smaller than the others (21). The caudal border is defined in the EORTC guidelines as 15 mm above the penile bulb or at the apex of the prostate whereas FROGG and PMH guidelines suggest that it should be 5–6 and 8–12 mm below the VUA, respectively. For RTOG group, the inferior treatment volume should end immediately superior to the penile bulb and it employs sagittal reconstruction to identify the most inferior urine in the bladder. At mid plan, all four consensus advocate the region extending anteriorly to posteriorly from the pubic symphysis to the rectum should be included. The superior border is also controversial following these guidelines: FROGG guidelines suggest the volume encompassing the entire SV bed and distal portion of the vas deferens; the bladder neck for EORTC guideline; the superior surgical clip or 5 mm above the vas deferens for PMH guideline; and the level of the cut end of vas deferens or 3–4 mm above the top of the symphysis in the RTOG guideline. The EORTC does not include the bladder in its CTV definition while the RTOG, FROGG, and PHM groups include 1.5 cm of posterior bladder and bladder wall. A special focus seems interesting with the recommendations to include or not the SV bed following VS invasion or not (pT2–pT3a/pT3b): for RTOG guideline, the VS bed should be delineated based on surgical clip visualization or VS remnants partially in case of pT2 or pT3a at apex and pT3a at the base or involvement of VS required inclusion of the SV remnants totally; for PHM guideline in case of pT2–pT3a, the superior boundary is the superior surgical clip or 5 mm above the inferior border of the vas deferens and retained VS are included in case of pT3b but with 1 cm extension beyond the gross recurrent disease; for FROGG guideline, pT2–pT3a case should include VS bed and any residual VS should be included in CTV delineation in case of pT3b; and for EORTC guideline, the site of the base of VS should be included in any case with a 5 mm in all directions to account for microscopic extension, with the original location of VS in case of pT3b.

**Table 1:** **Description of consensus guidelines**.

Protocols/boundaries	Princess Margaret Hospital	European Organization for Research and Treatment of Cancer (EORTC)	Faculty of Radiation Oncology Genito-Urinary Group (FROGG)-ANZR	Radiation Therapy Oncology Group
Superior	Superior surgical clips if present, or 5 mm above the inferior border of the vas deferens. Retained seminal vesicle (SV) included when pathologically involved	Bladder neck +5 mm in all directions Original site of the base of SV should be included. If SV involved, include original position ± the remnants	Encompass all of the SV bed as defined by non-vascular clips and should include distal portion of the vas deferens. If SV pathologically involved, include any residual SV	Level of cut end of vas deferens or 3–4 cm above top of symphysis. Include SV remnants if pathologically involved
Inferior	8 mm below the vesicourethral anastomosis (VUA) or the top of the PB, whichever is most superior	Apex −15 mm cranially from the PB +5 mm in all directions	5–6 mm below the VUA, but should include all surgical clips inferiorly. If VUA not clearly defined, then slice above the PB	8–12 mm, below VUA, may include more if concern for apical margin. Can extend to slice above PB if VUA not well visualized
Lateral	Caudal: medial border of the levator ani and obturator internus. Cranial: sacrorectogenitopubic fascia	Up to the neurovascular bundles (if removed up to the ilio-obturatic muscles) + 5 mm in all directions	Medial border of the levator ani muscle or obturator internus muscle	Below superior edge of symphysis pubis: levator ani muscles, obturator internus Above superior edge of symphysis pubis:sacrorectogenitopubic fascia
Anterior	Caudal: posterior edge of the symphysis pubis up to the top of the symphysis pubis. Cranial: posterior 1.5 cm of the bladder wall	Anastomosis and urethral axis +5 mm in all directions	Lower border of clinical target volume (CTV) to 3 cm superior, posterior aspect of the symphysis pubis. More superiorly: posterior 1.5 cm of the bladder	Below superior edge of symphysis pubis: posterior edge of pubic bone. Above superior edge of symphysis pubis: posterior 1–2 cm of bladder wall
Posterior	Caudal: anterior border of the rectal wall and levator ani. Cranial: mesorectal fascia	Up to but not including the outer rectal wall, cranially including the most posterior part of the bladder neck +5 mm in all directions	Levator ani and anterior rectal wall. More superiorly, anterior mesorectal fascia	Below superior edge of symphysis pubis: anterior rectal wall Above superior edge of symphysis pubis: mesorectal fascia
CTV (cm^3^)	104 ± 25	60 ± 17	88 ± 16	102 ± 24

### CTV Delineation Using Multiparametric MRI

The previous four consensus guidelines were published considering CT as reference imaging system except for PHM guideline that used postoperative MRI to contribute in CTV delineation if local recurrence was detected. Multiparametric MRI scans (T2-weighted and dynamic contrast-enhanced images) have been shown to be an effective tool for evaluation of the prostatic fossa and to detect local recurrence (11, 22). As published guidelines propose to include the cut end of the vas deferens (RTOG, PHM) as a distinct postoperative feature, this organ is visible on MRI (22). Furthermore, postoperative findings of the SV are highly variable, and it has been showed by Sella et al. that in a postoperative MRI study, 20% of the patients had SV remnants, with similar location of the preoperative SV position, with an additional 38% with fibrotic SV tips (10). In most European countries, a preoperative MRI is not still routinely carried out as part of the workup before RP. Croke et al. looked on 20 patient candidates for postoperative RT whose preoperative staging MRIs were fused with postoperative planning CT scans on whom the 4 CTV delineation guidelines had been applied previously. In all the 20 cases, the CTVs from guidelines did not cover the MRI-defined prostate generating an average prostate volume geographic miss of 35% (23). A second study on 30 patients analyzed CTVs contoured from RTOG Consensus guidelines (CTV RTOG) to CTV based on preoperative MRI (CTV MRI). CTV MRI was a mean of 18.6% larger than CTV RTOG with a mean volume of 138 cc versus 116.3 cc, respectively (24). On 10 patients MRI-detected biopsy proven local tumor recurrence with postprostatectomy prostate cancer, Wang et al. showed that in the superoinferior direction, recurrences ranged from the superior retrovesical region, to the inferior retrovesical region, to the posterior anastomosis, and as inferiorly as the posterior urogenital diaphragm. They reported that RTOG CTV contours did not appear adequate posterolaterally near the rectum/mesorectal fascia and at the posterior urogenital diaphragm inferiorly (25).

Potential benefit of postoperative MRI is to enable a clinician to better delineate areas of identified local recurrence. Based on a study of 113 patients diagnosed with prostate cancer recurrence by MRI scan, Park et al. showed that almost 95% lesions were located within 10 mm of the midline. With the use of the inferior border of the pubic symphysis as a reference point they showed that 87.3% lesions were located within 30 mm in the cranial direction from the reference point (12). For pT2–pT3a patients, VUA site and bladder neck represented most recurrence locations whereas for pT3b patients VUA site and retrovesical area were predominant. Hence, the authors recommended optimal CTV guidelines based on the pattern of local recurrence detected with an MRI acquired before salvage RT (SRT) and they displayed a CTV suggestion encompassing 97% of suspected tumor recurrences (representing a mean CTV volume of 15 ± 5 cm^3^). A similar study was conducted by Miralbell et al., and they suggested a 4 × 3 cm sized, cylindrically shaped CTV, centered 5 mm posteriorly and 3 mm inferior to the VUA site (11).

## Ongoing Trials for Postoperative Prostate Cancer Radiotherapy

Radiotherapy might have a meaningful benefit after RP, but there are no good data on the optimum timing of RT (26). A policy of adjuvant RT would result in significant overtreatment, while an early SRT policy might be equally effective. Likewise the optimum duration of HT combined with RT after RP is an important issue. Hence, different collaborative groups worldwide have started randomized controlled trials (RCT) to assess the benefit of RT ± HT and its best timing between adjuvant or salvage settings (see Table 2). These prospective studies are currently using a delineation policy following one of the already published guidelines.

**Table 2:** **Overview of ongoing phase III studies on postoperative RT**.

Protocols	Randomization/RT dose	Guidelines used in trials				
RAVES (NCT00860652)	ART commenced at ≤4 months of RP or early SRT triggered by a PSA level of >0.20 ng/mL RT dose 64 Gy	FROGG guidelines				

Radiation Therapy Oncology Group (RTOG) 0534 (NCT00567580)	SRT with or without HT 6 months or pelvic fields RT dose 64.8–70.2 Gy prostate bed and 45 Gy pelvic lymph node	RTOG guidelines				

GETUG-AFU 17 (NCT00667069)	ART vs SRT with 6 months HT RT dose 66 Gy/33 Fract	GETUG guidelines				
		Superior	Inferior	Lateral	Anterior	Posterior
		Include VUA, bladder neck and prostate fins laterally. 4.5–5 cm above penile bulb, fatty space between bladder and rectum is delineated. In case of SV invasion or pT3a at prostate base, SV bed should be included on 1,5–2 cm high with rectum wall to be spared	5–10 mm above the penile bulb	Medial border of the levator ani muscle	To posterior part of cavernous corpus to 1/3 superior zone of pubic symphysis and bladder neck	From anal canal to anterior rectal wall and mesorectal fascia with a posterior limit following prostate fins

RADICALS (NCT00541047)	First randomization: ART or SRT Second randomization: RT only/RT + HT 6 months/RT + HT 24 months RT dose 66 Gy/33 Fract or 55 Gy/20 Fract	RADICALS guidelines modified from Wiltshire and colleagues

EORTC 22043 (NCT00949962)	ART or SRT with or without HT 6 months	EORTC guidelines

SAKK 09/10 (NCT01272050)	SRT with RT dose 64 or 70 Gy	EORTC guidelines

MAPS (NCT01411345)	SRT with or without boost RT dose 68 Gy or 74.8 Gy/34 Fract	–

*ART, adjuvant RT; SRT, salvage RT; RP, radical prostatectomy; PSA, prostate-specific antigen; HT, hormonal treatment; VUA, vesicourethral anastomosis; SV, seminal vesicle; Fract, fractions; GETUG, Groupe d’Etude des Tumeurs UroGénitales; RT, radiotherapy*.

In France, GETUG members set up a meeting with 12 radiation oncologists held on 5 March 2008 for contouring session in postoperative prostate cancer. These results were published in French for CTV delineation (27) and atlas (28) and a report of the GETUG guidelines are listed in Table 2. This workshop helped to create CTV planning for the already published GETUG-AFU 16 study (29) and for the ongoing GETUG-AFU studies: GETUG-AFU 22 (questioning RT vs RT + HT for early SRT, NCT01994239) in a phase II study and GETUG-AFU 17 (adjuvant versus salvage treatment with a combination of RT and HT, NCT00667069) in a phase III study.

Interestingly, the important number of RCT could validate guidelines used as reference for contouring in a prospective setting and might help clinicians to choose among these protocols the best to cope with postoperative RT.

## Discussion

The debate to promote adjuvant or SRT is still an important issue and prostate bed target delineation remains in this context difficult as location and size of recurrences can be different (i.e., being macroscopically detectable by MRI) with time from PR. Considering the four published guidelines, anyone should be aware that despite different methods used some aspects remained similar and others showed discrepancies especially in volume delimitations, including also GETUG guidelines (17–20). Moreover, definition of recurrences to limit target contouring from these guidelines comes from macroscopic imaging description, generally assessed lately in the history of postoperative RT indication (30). These discrepancies explain why there are so different CTV volumes among these four guidelines and a comparison of theses four consensus has been carried out in a Canadian study (16). For each patient of the 20 treated in this study, a CTV delineation following these four guidelines was performed and analyzed. Results showed that EORTC-CTV covered a larger volume of normal tissue posteriorly (more rectal volume) than the other guidelines. A greater coverage of the bladder was noticed for RTOG/PMH-CTV compared to EORTC-CTV (21). The inherent difficulty in defining the “virtual” prostate bed target is reflected in the presence of interobserver variability in the delineation of the prostate bed that appears to persist even despite the use of rigorous contouring protocols and guideline (31, 32). Ost et al. assessed interobserver agreement (six observers participated in this study) of prostate bed delineation using CT alone as proposed by EORTC guidelines and found a moderate agreement with an overall standard deviation of the outer margins ranged from 4.6 to 7 mm (31). For Symon et al., 38 pfCTV were delineated on postradical prostatectomy CT scans of 8 patients by 5 observers. Interphysician variability was considerable with a mean pfCTV of 39.09 cm (range, 11.8–72.5 cm). pfCTV delineation was subject to considerable interobserver variability associated with a significant risk of inadequate targeting of the anastomosis/bladder neck region and the retrovesical space (32).

The validation of existing consensus through ongoing clinical trials arose the need for high quality assurance (QA) program. The practice of dummy run (DR) in RCT has already be shown as an efficient tool for QA optimization (33). In order to assess the compliance to the 3D-CRT protocol guidelines, 30 participating centers were requested to participate in a DR procedure for the EORTC trial 22991 and patients files harbored no major protocol deviation (34). The SAKK 09/10 study including a site-specific and study-specific questionnaire and a DR, following EORTC contouring guidelines. In the first submitted version of the DR, major deviations were noted for 70% of the centers. These results were improved after DR completion for 83% of the centers in this study. A moderate interobserver agreement was noticed in prostate bed delineation initially, and DR protocol achieved to improve the acquaintance of the participating centers with the trial protocol (35).

Education can be useful to correct for existing discrepancies and to drive professionals toward a harmonization of practice. Mitchell et al. had showed that interclinicians variability in target volume outlining existed but adherence to evidence-based protocol (RADICALS protocol) can achieve reduction in this variability (36). Pasquier et al. conducted in 11 RT centers a prospective work to improve homogeneity of delineation of volume of interest on 3 clinical cases of which a case for a postoperative prostate cancer (37). After collecting each initial delineated volume and comparing these volumes with validated indexes [volume ratio (VR), volume overlap, and Dice similarity coefficient (DSC)], a second delineation was secondly performed after discussion of the slice results. For the selected case, VR and AV were significantly improved and DSC remained high, so the authors showed that a collaborative discussion about clinical case and the choice of shared guidelines (RTOG in this article) could improve the homogeneity of CTV delineation (37). Another study from the same team analyzed automated atlas-based segmentation supplied by software vendors compared to radiation oncologist contours for prostate bed cases. They showed that these algorithms for segmentation were essentially aimed to delineate OAR (high-contrast organs) and were insufficient for prostate bed contours (38).

## Conclusion

Adjuvant or SRT in the era of 3D-CRT or IMRT are based on optimal contouring methods to avoid geographic miss in an invisible target. Delineating the prostate bed remains an issue as current CTV consensus definitions do not adequately cover the prostate bed and/or GTV based on preoperative imaging. These published guidelines based on postoperative CT ± MRI imaging, even if discrepancies between them exist, have played an important role for professional support. Adopting one of them as a standard of care in its own practice provide better delineation homogeneity and best covering of the entire surgical bed in postoperative radiotherapy. This statement might be relevant as long as ongoing clinical trials carry new standard of care and might emphasize which guideline offer the most appropriate long-term local control following postoperative prostate cancer radiotherapy with an acceptable toxicity profile.

## Author Contributions

IL wrote the manuscript, reviewed it, and sent it to the correspondent author; responsible for charts. PS reviewed the manuscript and made corrections. GL, SS, SG, and CC reviewed the final manuscript. SS is in charge for topic selection.

## Conflict of Interest Statement

The authors declare that the research was conducted in the absence of any commercial or financial relationships that could be construed as a potential conflict of interest.
